# Drug discovery oncology in a mouse: concepts, models and limitations

**DOI:** 10.2144/fsoa-2021-0019

**Published:** 2021-06-23

**Authors:** Jason E Long, Maja Jankovic, Danilo Maddalo

**Affiliations:** 1 Department of Translational Oncology, Genentech, Inc., South San Francisco, CA 94080, USA; 2 Department of Medicine, Division of Experimental Medicine, McGill University, Montréal, QC, H4A 3J1, Canada; 3 Lady Davis Institute for Medical Research, Montréal, QC, H4A 3J1, Canada; 4 Pharmaceutical Research & Early Development, Roche Innovation Center Basel, F. Hoffmann-La Roche Ltd, Basel, 4070, Switzerland

**Keywords:** drug discovery, *in vivo* CRISPR/Cas9, mouse models, SEMMs, translational cancer research

## Abstract

The utilization of suitable mouse models is a critical step in the drug discovery oncology workflow as their generation and use are important for target identification and validation as well as toxicity and efficacy assessments. Current murine models have been instrumental in furthering insights into the mode of action of drugs before transitioning into the clinic. Recent advancements in genome editing with the development of the CRISPR/Cas9 system and the possibility of applying such technology directly *in vivo* have expanded the toolkit of preclinical models available. In this review, a brief presentation of the current models used in drug discovery will be provided with a particular emphasis on the novel CRISPR/Cas9 models.

## Lay abstract

Picking the right animal model is a critical step in the drug discovery oncology workflow. Current murine models have provided a key contribution to the advancement and refinement of drugs before transitioning into the clinic. The application of the CRISPR)/Cas system as a tool to genetically engineer preclinical models based on genomic information has revolutionized the field by reducing the cost and the time and while increasing versatility. In this review, the authors will provide a brief overview of preclinical models employed in drug discovery, with particular attention to the ones generated with the CRISPR/Cas9 technology.

Development of clinical therapeutics in oncology is a costly and labor-intensive process. It is often fraught with successive failures until a suitable candidate is discovered that can progress into a clinical trial for human patients. Once a clinical trial has been initiated, the odds of approval success are around 10%, with an ultimate cost of approximately $1 billion and a decade’s worth of work [1,2]. The predominant issue that all potential medicines face during the drug development process is the disparity between the results obtained from clinical trials and the results from preclinical experiments. Even those therapeutics that successfully make it through tend to have less than stellar response benefits, generally ranging from 4 to 20% or eventually develop acquired resistance over the course of treatment [3–5]. The goal of scientists working in the preclinical arena is to deliver a potential medicine with the highest chance for success while mitigating toxicity or undesirable or uncontrollable side effects that would cause the drug to fail. Preclinical *in vitro* and *in vivo* models play a pivotal role as a translatability bridge for this observed gap in results. Choosing the best model(s) during the discovery process for a given indication will provide a greater chance of success for scientists developing therapeutics. A summary of commonly used preclinical models used in oncology research is provided in Table 1.

**Table 1.** Comparisons of *in vivo* cancer models and their applications.

Model	Advantages	Disadvantages	Applications
Xenograft	• Cost efficient • Time efficient • Consistent genetics	• No intact immune system • Homogeneous	Early target validation; PK/PD correlation
PDX/PDOX	• Patient material • Expandability • Heterogeneous • Time efficient	• No intact immune system • Genetic drift over time • Cross species interaction	Efficacy and indication expansion at late stage of development
Syngeneic	• Intact immune system • Consistent genetics • Cost efficient • Time efficient	• Homogeneous • Steep growth • Limited response • Limited models available	Immuno-oncology related questions
GEMMs	• Intact immune system • Tumors are *in situ*	• Imaging required • Limited tumor mutation burden	In-depth investigation of mechanism of action
SEMMs	• Cost efficient • Time efficient • Tumors are *in situ* • Intact immune system	• Limited by organ accessibility • Imaging required • Limited mutation burden	In-depth investigation of mechanism of action *In vivo* target identification
Humanized mouse models	• Intact immune system • Directly from patients	• Time/resource intensive • Graft-vs-host disease	Immuno-oncology related questions

GEMM: Genetically modified mouse model; PD: Pharmacodynamics; PDOX: Patient-derived orthotopic xenograft; PDX: Patient-derived xenograft; PK: Pharmacokinetics; SEMM: Somatically engineered mouse model.

Choosing a model during the drug discovery process requires a multifaceted approach. As the spectrum of key scientific questions during the drug discovery process is rather broad, a single preclinical *in vivo* model would provide just partial information. For this reason, the comparison between *in vitro* and *in vivo* models, as well as among *in vivo* models, is often misleading as it implies the differentiation between better or worse models. However, the suitability and use of such models should be dictated by the scientific question being asked. In addition, when selecting, designing or generating preclinical models, the level of translatability into patients should be considered based on target similarity, mechanism of action and other noncell autonomous factors involved between human and mouse. This is even truer today given that the drug discovery field is increasingly moving toward personalized therapies and away from universal chemotherapy-based medicines.

However, despite genetic similarities of approximately 80% between commonly used mouse model species and humans, subtle differences in gene families, duplications and intricate gene regulation can have a dramatic impact on therapeutic development [6]. This issue is further complicated not only by the aforementioned genomic differences, but also by the complex biological systems of an organism such as gastrointestinal microbiota [7–9], hormonal regulation [10–12] and stromal and immune compartments [13–15]. Historically, the therapeutic workflow has relied on a limited number of model systems, each providing partial information on the mechanism of action, efficacy and toxicity of a drug. Lengthy timelines and the relative high cost for generating new target-relevant models have placed barriers to adding them to the drug discovery workflow. Fortunately, recent revolutionary advances in *in vivo* genome editing allow scientists to rapidly generate advanced mouse models, vastly increasing the ability to test specific disease indications and the accompanying genomics with closer fidelity to the human condition. Herein, we discuss some of the various types of currently available mouse models of cancer impacted by these rapidly evolving genome editing techniques. A summary of the preclinical animal models used in oncology drug discovery is provided in Figure 1.

**Figure 1.**
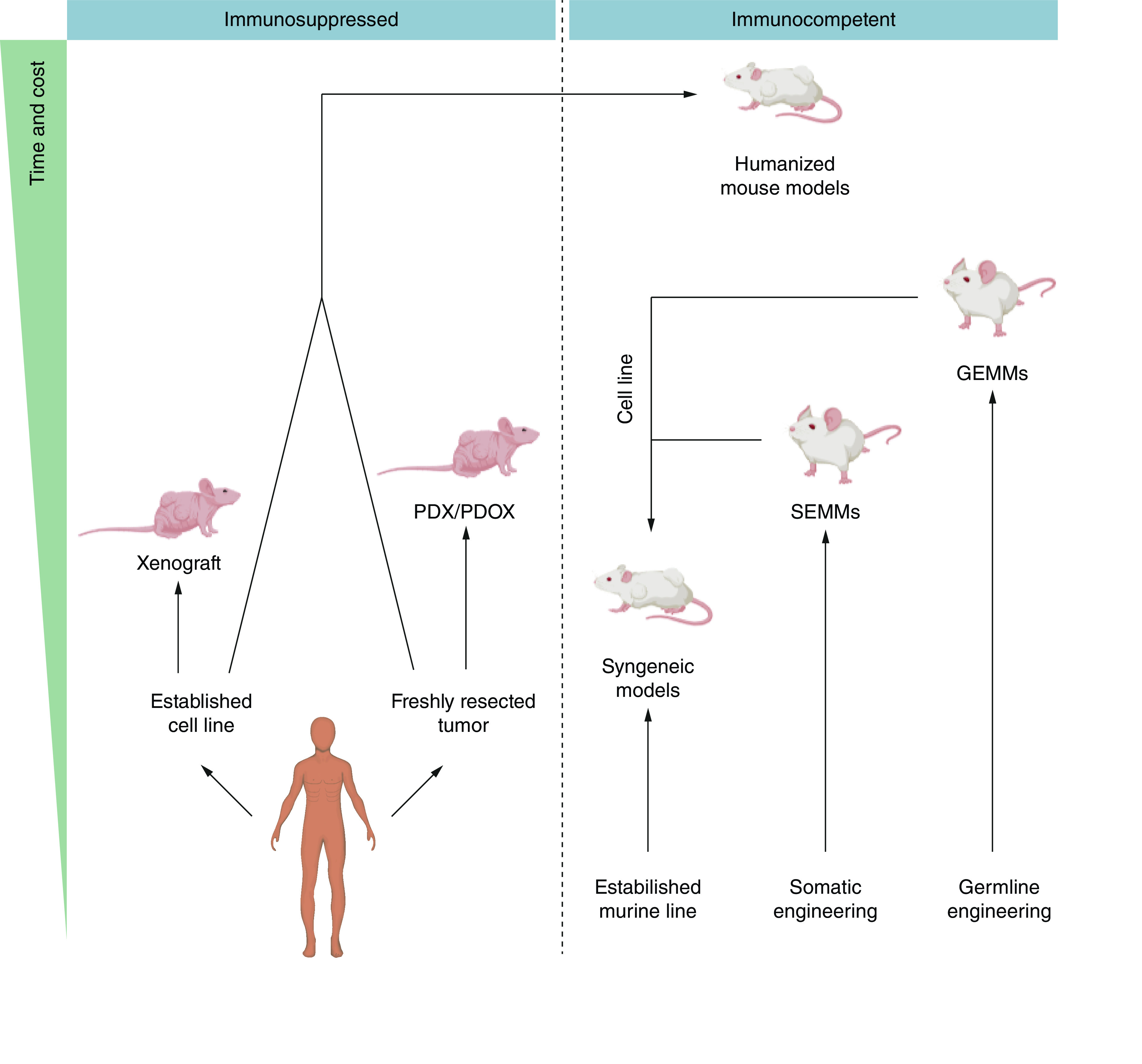
Schematic representation of animal models in drug discovery. GEMM: Genetically modified mouse model; PDOX: Patient-derived orthotopic xenograft; PDX: Patient-derived xenograft; SEMM: Somatically engineered mouse model.

## Xenografts

The most widely used *in vivo* systems employed in drug discovery are represented by xenograft models, where a previously established immortalized cancer cell line is injected subcutaneously into an immunocompromised mouse [16]. There are three predominantly used mouse lines used for these types of studies. In order of their level of immunodeficiencies, first are nude mice, which have severely reduced numbers of T cells due to a severely deteriorated or absent thymus. The second are nonobese diabetic severe combined immunodeficiency mice, which do not contain functional T or B cells. And at last, a variation of the nonobese diabetic severe combined immunodeficiency mice with an additional mutation in the IL2Rγ, which causes a lack of functional natural killer cells [17–19]. The use of these immunocompromised mice prevents the tumor cells from being rejected by the host’s immune system.

As a result of the subcutaneous implantation site, tumors are easily accessible to follow growth and/or response via calipers, fluorescence or bioluminescent imaging, as well as to measure drug concentration. For this reason, such an approach has allowed for quick validation of target dependencies and enabled preliminary studies investigating pharmacokinetics (PK) and pharmacodynamics (PD) parameters of candidate therapeutics. Studies can be run with xenografts in a reproducible and efficient manner as the cultured cells are grown *in vitro*, collected and uniformly injected into animals that are genetically similar. As the establishment of this mouse model is highly reproducible, requires the least investment in terms of time and money and the tumor mass can be easily isolated from the host, xenografts have been successfully integrated into the path of drug discovery and represent the most used mouse model in the field (Figure 1).

Despite their vast application, cell implantation into immunocompromised animals has a few intrinsic caveats. First, xenografts are highly dependent on the ability of the tumor cells to successfully grow subcutaneously, which is usually outside of their original microenvironment. However, dependent on the cell line, if the growth rate is excessive, the tumor’s structure can be affected by ulceration on the outer surface and/or necrosis of the inner mass, potentially impacting the experimental readout [20]. Some of these limitations can be overcome by implanting the tumor cells into their tissue of origin [21]. A small caveat using these long-term established cell lines is their limited representation of the full genomic range of a disease indication. A more considerable concern is that xenograft models are established in animals lacking a fully functioning immune system, precluding their use for those investigating noncell autonomous targets. Furthermore, large scale investigations of the translatability of xenograft studies have yielded poor or variable correlations [22–25]. Conversely, if gene expression analyses are used on available cell lines, rather than their tissue type of origin, to match a specific disease indication, the results are more favorable [26–28].

## Patient-derived models: patient-derived xenografts & patient-derived orthotopic xenografts

Cell-based xenograft models often offer limited patient tumor representation and considerable efforts have been made to develop a similar, yet more representative disease system. These efforts have yielded patient-derived xenografts (PDXs), which are generated using freshly resected tumor fragments implanted subcutaneously into immunocompromised animals (Figure 1) [16,29–32]. These tumors can then be grown, resected and re-implanted in additional animals to analyze tumor growth, evolution and responses to therapy. Large databases of PDXs have been assembled by consortia in Europe (http://www.europdx.eu/) and the USA (http://www.pdxnetwork.org/) to help researchers by providing valuable sequencing information and standardization protocols for their use. Current PDXs offer the ease of use of a xenograft system, but with the distinct advantage that tumors are obtained directly from a patient and contain the genomic, stromal makeup and extracellular matrix fingerprints of the disease [33–37] with higher fidelity over multiple passages than cell line-based xenografts [38–40]. In addition, PDXs are an extremely valuable tool to validate biomarkers to predict patient response to therapy and stratification.

One caveat to PDXs as a model system is the ability of the tumors to grow subcutaneously. Their take rate is highly variable, ranging from 5–60%, dependent on both the type and stage of disease tissue [29,41–44]. To help alleviate this problem, orthotopic models of patient-derived orthotopic xenografts (PDOXs) have been developed [45–53], where the tumor is surgically implanted into the same organ of the mouse from which it was resected in the human. These procedures do impact the throughput of the model and imaging of tumor growth kinetics, but offer a better recapitulation of the tumor microenvironment and a better understanding of metastatic mechanisms involved in the disease [52]. Another major advantage of PDXs is their use as ‘avatars’, where the patient’s tumor can be evaluated in real time for therapeutic response and resistance [54]. Unfortunately, repeated passaging of patient tumors results in an increased growth rate that correlates with increased tumor grade [55]. However, the increased use of PDXs with various therapeutics should provide a wealth of knowledge for future treatment options.

Although PDXs are closer to recapitulating human disease by including the accompanying human stroma, this stroma, mainly fibroblasts, is slowly replaced by the mouse equivalents over a short time. This change can have potential complications with interpreting therapeutic response, but it should be noted that the engrafted tumor cells preserve the ability to recruit stroma to tumor microenvironment [54,56]. Furthermore, it has been shown that various growth substrates used for implantation, such as Matrigel (Corning Life Sciences, NC, USA), have differential impacts on cell signaling and hormonal response [57]. In addition, PDXs share some of the shortcomings as the xenografts, namely they are established in immunocompromised animals to avoid rejection of the human tumor, and are therefore not amenable for studies concerning immune-based targets.

## Syngeneic models

The recent renewed interest in immune checkpoint and costimulatory molecules for treating cancer has encouraged many researchers to revisit one of the oldest mouse model systems, the syngeneic mouse model [58–60]. Syngeneic models make use of cell lines typically derived from either spontaneous tumors or those arising due to chemical treatment or viral integration. The reason is that, in this model system, the tumor cells, tumor microenvironment and host all have the same genetic background, which allows the mice to have a competent immune system. Therefore, since tumor rejection will not occur, therapeutic impacts on tumor growth, metastasis and immune modulation can be thoroughly studied in a complete system. Like xenografts, syngeneic studies can be performed in statistically significant numbers relatively easily due to the use of *in vitro* cultured, immortalized cell lines that are subcutaneously or orthotopically implanted. These cell lines have been generated in a variety of inbred mouse strains, commonly C57BL/6, BALB/c or FVB mice [59].

However, one pitfall to the syngeneic models is that different results can be obtained dependent on the inbred model background chosen. This is likely due to slight variances in the immune infiltrate, husbandry facilities, genetic drift within a strain over time, retrotransposon activity and molecular pathway differences [61–67]. Similar to xenografts, implanted tumor growth rates can be much faster than spontaneously occurring tumors, leading to undesired tumor effects such as necrosis and immune effects such as the strong correlation of immune infiltrate loss with increased tumor size [68]. Another concerning observation has shown that metastases tend to be implant site dependent and that orthotopic tumors have a higher probability of maintaining stromal characteristics than tumors implanted subcutaneously [58,69], so great care must be undertaken when designing these studies.

## Somatically engineered mouse models

The recent development of the CRISPR/Cas9 system has made genome editing an affordable and feasible technology for *in vitro* as well as *in vivo* applications. In order to efficiently and precisely induce double-strand breaks in the genome, the CRISPR/Cas9 system requires the codelivery of the Cas9 nuclease and a 20-nucleotide long single guide of RNA (sgRNA) complementary to the target region. Induction of a double-strand break by Cas9 activates the nonhomologous-end-joining repair pathway that eventually introduces insertions or deletions (indels) at the target site. The straightforwardness and efficiency of the technology have prompted the generation of several animal models by direct genome editing of germline, as well as somatic cells. In general, *in vivo* genome editing with the CRISPR/Cas9 system can be achieved by delivering the system in one of three ways: as viral particles, as naked DNA or as a ribonucleoprotein complex. Since a limiting factor may be the size of the Cas9 protein (>4 kb), mouse models have been engineered to express the endonuclease conditionally [70–73] or constitutively [72].

Generation of somatically engineered mouse models (SEMMs) by direct genome editing of somatic cells has several advantages [74]. First of all, as no embryonic stem cell (ESC) engineering is required, timelines are often reduced, as well as the cost for the generation of each model. In addition, different genetic lesions can be easily generated by simply swapping sgRNAs. Alternatively, oncogenic signatures shared in different organs can be induced by changing the site of injection. Another advantage of CRISPR-based models is their application to different genotypes. The most relevant example is the use of pre-existing Cre/LoxP-based genetically modified mouse models (GEMMs) to activate the oncogene *KRas^G12D^* and delete the tumor suppressor *Trp53* and subsequently, knock out additional tumor suppressors like *Nkx2.1*, *Pten* and *Apc* using the CRISPR/Cas9 system [75]. The difference in impact of the loss of function of particular genes during lung cancer development was able to be interrogated with minimal time and cost investment. In addition, the same system was used in a KRas-driven pancreatic cancer model to show that loss of *p57* leads to insensitivity to combined inhibition therapy of BET family proteins [76]. In the same year, Chiou *et al.* generated a Cre-dependent conditional Cas9 knock-in mouse, which was then crossed to a conditional KRas^LSL-G12D^/+ mouse. They demonstrated through pancreatic retrograde delivery of a lentivirus expressing Cre-recombinase and a guide against *Lkb1* (sgLkb1) that loss of function of LKB1 leads to advanced pancreatic tumorigenesis [77].

By combining CRISPR/Cas9 based screenings and metabolomics, Romero *et al.* introduced a novel KRas-driven lung cancer model in which they showed that loss of Keap1 resulted in a genotypic specific glutamine dependency. Furthermore, they showed that KEAP1/NRF2 mutant cancers could be therapeutically exploited by the pharmacological inhibition of glutaminase [78]. The Zhang lab induced targeted gene disruption using Cas9 and guide RNAs by using adeno-associated viral delivery to target single (*Mecp2*) as well as multiple genes (*Dnmt1*, *Dnmt3a* and *Dnmt3b*) in the hippocampus [79] and liver [80].

In addition to altering a single gene, a CRISPR-based approach also provides the possibility of inducing chromosomal rearrangements like deletions, inversions and translocations by co-expressing two sgRNAs. The first example of such an approach was provided by delivery of an all-in-one adenoviral vector expressing Cas9 and two sgRNAs targeting the intronic region of the genes *Eml4* and *Alk* on mouse chromosome 17. Upon delivery of this adenovirus, the oncogenic chromosomal inversion EML4-ALK was induced directly into the mouse lung epithelium, generating non-small-cell lung cancer rapidly in 4 weeks post infection, which would eventually progress into an adenocarcinoma within 8 weeks [81].

Another approach to creating somatic mutations for cancer modeling is the *ex vivo* modification of stem or progenitor cells that can then be transplanted into a xenograft model. For instance, the Ebert laboratory made use of CRISPR/Cas9-based multiplexed gene targeting of hematopoietic stem cells to identify multiple combinations that drive leukemogenesis [82]. Another study by Roper *et al.* utilized *in situ* gene editing to generate colorectal cancer models to be eventually transplanted orthotopically into recipient animals, resulting in an improved colorectal cancer model with enhanced engraftment and straightforward tumor formation [83].

CRISPR-based models also afford the option to introduce specific mutations by providing a DNA sequence (single or double stranded) to be used as a repair template in the target region by homologous directed repair (HDR) mechanisms. Pioneering work by Xue *et al.* demonstrated multiplexed CRISPR/Cas9-based somatic gene editing in the liver by using hydrodynamic tail injections of naked CRISPR/Cas9 plasmids with two sgRNAs against *Pten* and *Trp53* [84]. The CRISPR-mediated *Pten*- and *p53*-mutated mice showed histological hepatocellular carcinoma, which was in line with pre-existing tumor formation in double-transgenic *Pten^fl/fl^;Trp53^fl/fl^* mice. Moreover, they showed that singly targeted PTEN or TRP53 did not cause any detectable liver tumors, which was also observed in pre-existing transgenic *Pten^fl/fl^* and *Trp53^fl/f^*^l^ mice. These findings highlighted the ability of putative mutations by CRISPR/Cas9 in *in vivo* systems. Furthermore, in the same study they engineered gain-of-function mutations in the *Ctnnb1* gene (encoding for β-catenin) through HDR mediated gene correction, which introduced four point mutations in the serine/threonine residues of CTNNB1, thus preventing its degradation. *In vivo* genome editing mediated by HDR-induced mutation is however, much less efficient than a straight knockout model as the HDR pathway is active only in dividing cells. As a result, some models may lead to confounding data. For example, in a model of lung cancer, *Kras^G12D^; Trp53^-/-^; Lkb1^-/-^*, three sgRNAs and a donor template to induce the 12D mutation were delivered in an adeno-associated vector. As the HDR-mediated 12D mutation was less efficient than the knockout of *Trp53* and *Lkb1*, genotyping of many of the generated tumors showed a KRas-independent growth. In addition, a possibility exists of donor random integration and/or inclusion of part of the vector [85].

Since the experimenter can decide *a priori* the type of genetic signature to induce, SEMMs can also be used to identify/verify oncogenic driving properties of tumor suppressor genes and/or oncogenes, alone or in synergy. In addition, as tumors develop within their own microenvironment, these models are ideal for investigating noncell autonomous targets and tumor response. For this reason, it is very likely that these models will be gradually incorporated as additional tools to identify potential drug targets directly *in vivo*.

## Genetically engineered mouse models

GEMMs play a critical role in understanding tumor initiation and progression as well as resistance in specific microenvironments [86–88]. Genomic modifications by homologous recombination in ESCs have enabled the precise modification of oncogenes and/or tumor suppressor in the germline. However, sometimes models generated with this approach do not allow for long term studies as rapid tumor growth causes obstruction of the airways or GI tract, leading to early euthanasia.

In addition, manipulation of some tumor suppressors and/or oncogenes is embryonically lethal. Ideally a model closer to the real dynamics of tumor formation would involve a small subset of somatic cells in a specific tissue whose tumor progress goes through a similar disease staging as in humans. Tempo-spatial control of tumor formation has been achieved by introduction into ESCs of recombination systems at specific gene loci. Particularly the Cre/loxP or Flp/frt are mainly used to introduce conditional deletion of tumor suppressors, activation of oncogenes via a Lox-STOP-Lox cassette and chromosomal rearrangements. Germline modification to generate murine models of cancer provides a more complete scenario of tumor development and/or response to therapy as cancer develops in its site of origin in an immunocompetent animal, allowing investigation of tumor–stroma interaction.

Despite the relevance of the information provided, it has, however, been challenging integrating GEMMs into the drug discovery workflow because of the relatively high cost, the lengthy procedures required to manipulate mouse ESCs, as well as the long timelines required for the production of the colonies and the maintenance cost of genotyping and breeding [89,90]. As a general note, it has also to be considered that both GEMMs and SEMMs would require more complex tools to follow tumor growth and/or response to treatment such as μCT scan or MRI.

## Humanized mouse models

In recent years, the focus of drug discovery has gradually shifted toward the immune environment surrounding and infiltrating the tumors. Therefore, the need to include such information into an *in vivo* model has gained increasing importance. A cost- and time-efficient option is performed by the transplantation of total peripheral blood from human healthy donors or patients, usually peripheral blood mononuclear cells or in specific cases of the infusion of tumor-infiltrated lymphocytes [91–93]. In spite of their remarkable relevance in terms of disease/patient representation, this type of model is however limited by the development of severe graft-versus-host disease roughly 2–5 weeks after injection, giving therefore a narrow experimental window. To circumvent this limitation, several strategies aiming at knocking-in human components of the immune system sequentially have been developed [94]. A solution to delay or eliminate graft-versus-host disease development is modifying the germline of immunosuppressed animals by serial knock-ins of human genes regulating the immune system. While representing a remarkable improvement in the experimental conditions and helping understanding tumor–host interaction, this approach is limited by the presence of residual murine immune system (macrophages, dendritic cells and granulocytes) as well as by the species specificity of some cytokines.

A summary of advantages and disadvantage of the *in vivo* models in preclinical oncology drug discovery is provided in Table 1.

## The grey zone between *in vivo* & *in vitro*: tumor organoids

Ideally before investing in time-consuming *in vivo* approaches, the drug discovery workflow would benefit from an intermediate step where cells are organized in a 3D structure resembling the tumor mass. Recent advances in our understanding of stem cells and the conditions in which to propagate them have allowed for the development of a 3D *in vitro* model system [95–104]. Organoids can mimic some of the cell–cell interactions and functional aspects of a human organ, making it a useful model for answering specific questions where difficult access to the organ or slow tumor generation are a problem. Organoids are generated from pluripotent stem cells, either from the embryo (ESCs) or adult tissue, both normal and cancerous adipose derived stem cells (ADSCs). When these cells are grown in a 3D matrix, such as Matrigel or biologically/nonbiologically defined substrates [105–108], these cells can self-organize into complex structures. In order for the desired cell types and structures to form, a carefully orchestrated application of defined factors must occur [109,110]. The correct application sequence causes the cells of the organoid to develop spatial identity cues, facilitates terminal differentiation and allows for repeated 3D growth, all of which is necessary to recapitulate the human organ for disease modeling. Provided that the administration sequence or components required are known, organoids can be rapidly generated. Furthermore, they can be scaled up in large amounts for screening purposes and personalized as the cells are directly removed from the patient. Additionally, organoids have great accessibility for direct analysis and genetic manipulation. Unfortunately, as with some other models, there is a lack of different cell types such as the immune or stromal compartment, thereby giving an incomplete representation of the disease. One complicating factor is that organoids are somewhat costly to generate due to the requirement of specific cofactor molecules. Importantly, organoids may be heterogeneous even when comparing those derived from a single sample. This can be both detrimental due to inconsistent results and potentially very beneficial as the organoids can provide representation of all stages of a disease [111–114].

## Conclusion & future perspective

The wide spectrum of animal models available to model human cancer provide the possibility of tackling different key scientific questions but it has to be noted that a single *in vivo* system will always be far from providing a complete answer. It is ultimately the responsibility of the experimenter to understand and judge which animal model should be used in a case-by-case approach. Predicting efficacy and toxicology, as well as understanding the mechanism of action of drugs at the preclinical stage are key aspects of drug development and should always be considered within a more complex picture. Certainly, the possibility of editing the genome of somatic cells *in vivo* has offered the opportunity to implement a novel category of animal models into the pipeline (the SEMMs), adding variety to the *in vivo* pharmacology toolkit and paving the way for novel, direct *in vivo* functional genomics experiments. Some of the studies mentioned in this review are impressive examples of how CRISPR/Cas9-based somatic genome editing can be integrated into the therapeutic discovery workflow without replacing any of the pre-existing models, which are still a valuable source of information, especially in the context of studies involving pharmacokinetics and pharmacodynamics. In conclusion, we believe that the evolution of preclinical models will be a reflection of the evolution of novel medicines (e.g., cell and gene therapies) and the key questions raised during their therapeutic development.

**Executive summary**

- *In vivo* models in oncology are a key component for the drug discovery and development process.
- No model is a perfect fit throughout the drug discovery workflow.
- Each scientific question can be addressed with a specific *in vivo* model.
- The introduction of genome editing technologies such as the CRISPR/Cas9 system reduced cost and time for the generation of a broad range of preclinical models increasing agility.
